# Hypoglycemic and Hypolipidemic Effects of Polyphenols from Burs of *Castanea mollissima* Blume

**DOI:** 10.3390/molecules16119764

**Published:** 2011-11-24

**Authors:** Peipei Yin, Shan Zhao, Siyu Chen, Jieyuan Liu, Lingling Shi, Xinjie Wang, Yujun Liu, Chao Ma

**Affiliations:** National Engineering Laboratory for Tree Breeding, College of Biological Sciences and Biotechnology, Beijing Forestry University, Beijing 100083, China

**Keywords:** *Castanea mollissima*, tannins, polyphenols, diabetes, hypoglycemic, hypolipidemic, chestnut burs

## Abstract

Substantial evidence suggests that phenolic extracts of *Castanea mollissima* spiny burs (CMPE) increase pancreatic cell viability after STZ (streptozotocin) treatment as a result of their antioxidant properties. In the present study, the hypoglycemic and hypolipidemic activities of CMPE were studied in normal and STZ-induced diabetic rats CMPE were orally administrated at doses of 150 and 300 mg/kg twice a day for 12 consecutive days. Serum glucose, triglyceride, total cholesterol, HDL- and LDL-cholesterol levels, malondialdehyde (MDA) level and SOD activity in liver, kidney, spleen and heart tissues were measured spectrophotometrically. In normal rats, no significant changes were observed in serum glucose, lipid profiles and tissue MDA and GSH levels after orally administration of CMPE. In diabetic rats, oral administration of CMPE at a dose of 300 mg/kg caused significant decreases in serum glucose, triglyceride, total cholesterol, LDL-cholesterol levels, as well as MDA and GSH levels in spleen and liver tissues. However, the 300 mg/kg dosage caused a significant body weight loss in both normal and diabetic rats. The observed effects indicated that CMPE could be further developed as a drug to prevent abnormal changes in blood glucose and lipid profile and to attenuate lipid peroxidation in liver and spleen tissues.

## 1. Introduction

Diabetes mellitus is one of the most common endocrine metabolic disorders, characterized by hyperglycemia due to defects in insulin secretion, action, or both. Chronic hyperglycemia in diabetes is associated with long term damage, dysfunction and eventually the failure of organs, especially the kidneys, nerves, eyes and cardiovascular system, which has a significant impact on the health, quality of life, and expectancy of patients as well as on the health care system [1,2]. Although several approaches are presently available to reduce the hyperglycemia including insulin therapy, and treatment with sulfonylureas, metaformin, and α-glucosidase inhibitors, unfortunately, all of these therapies have limited efficacy and various side effects. Therefore, there is still an urgent need to search for new classes of compounds for the therapy of hyperglycemia [3]. In diabetes, hyperglycemia generates reactive oxygen species (ROS), which in turn leads to tissue damage, with lipid peroxidation, inactivation of proteins, and protein glycation as intermediate mechanisms for its complications such as retinopathy, nephropathy, and coronary heart disease [4,5]. Therefore, recently interest has focused on plant-based natural antioxidants such as tannins, polyphenols and flavonoids to reduce the negative effect of oxidative stress and free radicals in diabetes patients and to prevent the destruction of β-cells [6].

Chinese chestnut (*Castanea mollissima* Blume) is a wood plant widely cultivated in Europe, North America and Asia as an economic crop. Chestnut burs, 1 to 2 cm long, 1 mm thick spines, are usually discarded upon harvesting. Therefore, the use of chestnut burs as a potential source of pharmaceuticals and functional food ingredient is of great interest to the chestnut processing industries as a way of valorizing this waste product. Recently, Vázquez [7,8] found that the polyphenol extracts from burs of *C. sativa* exhibited antioxidant potential in DPPH (1,1-diphenyl-2-picrylhydrazyl), ABTS^+^ (2,2′-azinobis-3-ethylbenzothiazoline-6-sulfonic acid) radical assays and reducing power analysis. Similar results have been demonstrated in our lab where the phenolic extract from *C. mollissima* (CMPE) elicited antioxidant properties both in chemical antioxidant and cellular antioxidant analysis [9]. Mujić also reported that the spiny burs extracts of *C. sativa* could increase rat pancreatic β-cell viability after streptozotocin (STZ) treatment by protecting DNA from oxidative damage and by enhancing the natural antioxidant system *in vitro* [10]. Since diabetes is characterized by progressive pancreatic β-cell failure, the direct beneficial effects of phenolic-rich chestnut extracts on pancreatic β-cells indicated that the polyphenols of chestnut burs could be used as a potential pharmaceutical for diabetes. However, the potentially valuable effects of these chestnut extracts require further confirmation in experimental animals *in vivo*. The aim of this study was to evaluate the hypoglycemic and hypolipidemic activities of CMPE in normal and STZ induced diabetic rats.

## 2. Results and Discussion

### 2.1. Induction of Diabetic Models

**Figure 1:**
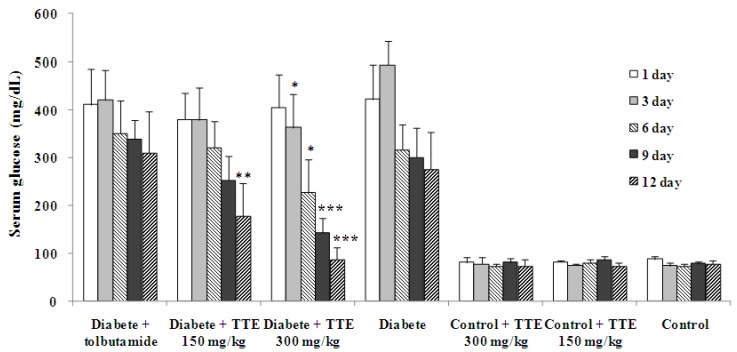
Effects of CMPE on fasting blood glucose levels in normal and STZ-induced diabetic rats. (Means ± SEM, n = 6). * p < 0.05, ** p < 0.01, *** p < 0.001, compared to diabetic rats on the same day.

STZ has been widely used intravenously or intraperitoneally (i.p.) to induce type I diabetes in animal models, especially rats and mice [11]. With a single i.p. injection of STZ at a dose of 60 mg/kg, as reported in the literature [6,12], the fasting blood glucose level of experimental rats showed a slight elevation, but a diabetic model could not be successfully established. Therefore, another injection of STZ at a dose of 60 mg/kg was given 5 days after the first one according to the literature [13]. A week after the second injection, almost all experimental animals showed significantly elevated fasting blood glucose levels (Figure 1). This phenomenon, together with polyphagia, polydipsia, polyuria and body weight loss, confirmed that the diabetic model had been successfully established. The reason for the failure of single injection might be related with the different strains of animals used, since different strains of animals are known to respond differently to STZ injection and different doses are needed for successful induction [14].

Tolbutamide is a first generation potassium channel blocker, sulfonylurea oral hypoglycemic drug, usually used in the management of type II diabetes if diet alone is not effective. However, to function it requires some pancreatic function, since the pancreas must synthesize insulin in order for this drug to work. Therefore, it is not effective in the management of type I diabetes [15]. As shown in Figure 1, oral administration of tolbutamide at a dose of 100 mg/kg didn’t elicit a significant decrease of fasting blood glucose level in diabetic animals, indicating that the pancreatic functions of experimental rats were severely damaged by STZ, which leads to a degeneration of the Langerhans islets beta cells [16,17] and the model established in this study was highly similar to type I diabetes. In some reports, tolbutamide showed significantly hypoglycemic effects in STZ induced diabetic rats [6,12], but these models were established with a single injection of STZ, and some remaining pancreatic functions may have contributed to the observed effects. 

### 2.2. Effects on Fast Blood Glucose Levels of Normal and Diabetic Rats

The effects of CMPE at dosages of 300 and 150 mg/kg on the fasting blood glucose levels of normal and diabetic rats are depicted in Figure 1. The vehicle-treated diabetic group showed a lower fasting glucose level after the 5th day compared to 1st day, indicating that experimental animals possessed limited auto-repair systems to partially repair the damage by STZ. Oral administration of CMPE at a dose of 300 mg/kg caused a significant decrease of fasting blood glucose level compared to control diabetic animals on the 3rd and 6th day (*p* < 0.05) and a very significant lower level on the 9th and 12th day (*p* < 0.001). This indicated that dosage of 300 mg/kg CMPE could effectively decrease the fasting blood glucose levels in diabetic rats. After treatment with CMPE at a dose of 150 mg/kg for 12 days, the fasting blood glucose levels of diabetics were also decreased very significantly compared to diabetic controls. Different from the effects on diabetics, no changes in fasting glucose levels were observed in normal rats after oral administration of CMPE both at a dose of 300 and 150 mg/kg, indicating that CMPE had no influence on fasting blood glucose levels of normal animals. Since the model established in this study was of type I diabetes, we may conclude that oral administration of CMPE for 12 days elicited a marked hypoglycemic effect in STZ induced diabetes. The *in vivo* resultsobserved here confirmed the report of Mujić, who found that the spiny burs extracts of *C. sativa* could increase rat pancreatic β-cell viability after streptozotocin treatment *in vitro * [10].

### 2.3. Effects on Body Weight of Normal and Diabetic Rats

The effects of CMPE on body weight of normal and diabetic rats were presented in Table 1. At the start of the experiment, there is no significant difference in body weight of vehicle treated diabetic rats compared to normal control rats. However, three days later, significant changes were observed, since significant body weight gain were observed in normal rats, while marginal body weight gain in diabetic rats. In normal rats, dosage of 150 mg/kg CMPE didn’t prevent body weight increase after 12 days treatment, while dosage of 300 mg/kg significantly attenuated the body weight gain. In diabetic rats, dosage of 150 mg/kg CMPE didn’t cause significant body weight changes compared to diabetic control group, but at a dose of 300 mg/kg, CMPE caused significant body weight loss after 9 days treatment. As a conclusion, at high dosage CMPE may attenuate the body weight gain in normal rats or decrease the body weight in diabetics. The effects observed here may be related with the physiological activities of tannins, which could bind with proteins and carbohydrates and lead to the formation of complexes rendering them undegradable [18,19], however, further studies are needed to clarify the mechanism in details.

### 2.4. Effects on Lipid Profile of Normal and Diabetic Rats

Regarding serum lipids, diabetes induction after 12 days caused significant increases in triglyceride, total cholesterol, LDL cholesterol and a significant reduction in HDL cholesterol concentrations compared to normal animals, as illustrated in Table 2. In diabetic rats, CMPE treatment at dose of 150 and 300 mg/kg for 12 days could significantly decrease the serum triglyceride, total cholesterol and LDL cholesterol levels compared to diabetic control groups. A dosage of 150 mg/kg also caused significant elevation of HDL-cholesterol levels in diabetic rats compared to diabetic controls. All these effects could significantly improve the lipid profile of STZ-induced diabetic rats. While in normal animals, no significant difference was observed between CMPE treated and vehicle treated groups for triglyceride, total cholesterol, LDL cholesterol and HDL-cholesterol levels. These results mean that CMPE treatment was able to improve serum lipid metabolites of diabetic rats, including decreasing the levels of triglyceride, total cholesterol, LDL cholesterol and increasing the level of HDL cholesterol. Usually, diabetes is associated with profound alternation in lipid and lipoprotein profiles as illustrated in this study and literature [20]. The results of this study showed that CMPE could reverse the hyperlipidemia in experimental diabetic rats, and thus may lead to a decrease in the risk of micro- and macrovascular disease and related complications [21]. The improvement of lipid profile might be directly or indirectly related with the reducing of blood glucose levels in diabetic rats.

**Table 1:** Effects of CMPE on body weight of normal and STZ induced diabetic rats.

Groups	Dose	Mean body weight ± SEM (g)					
	mg/kg	1st day	3rd day	6th day	9th day		12th day
Diabetes control	–	232.90 ± 17.84	238.78 ± 18.52 ^#^	237.73 ± 17.38 ^##^		239.20 ± 20.03 ^##^	236.80 ± 18.44 ^##^
Diabetes + Tolbutamide	100	244.66 ± 10.96	243.58 ± 11.50	239.52 ± 10.03		242.30 ± 8.15	237.76 ± 12.49
Diabetes + CMPE	150	244.05 ± 10.13	237.42 ± 7.18	231.05 ± 9.52		228.98 ± 10.84 *	222.98 ± 7.13 *
Diabetes + CMPE	300	240.07 ± 5.91	237.85 ± 9.01	224.40 ± 17.43 *		221.70 ± 19.05 *	218.50 ± 8.05 **
Normal control	–	236.32 ± 8.99	254.20 ± 10.10	270.17 ± 8.45		281.97 ± 8.38	300.17 ± 7.22
Normal + CMPE	150	238.55 ± 3.94	251.32 ± 4.59	269.83 ± 8.18		274.85 ± 5.64	283.50 ± 11.45
Normal + CMPE	300	232.72 ± 8.99	237.57 ± 10.10 ^#^	244.83 ± 8.45 ^#^		252.33 ± 8.38 ^##^	270.00 ± 7.22 ^##^

*n* = 6, SEM: Standard error of the mean. * *p* < 0.05, ** *p* < 0.01 significant from diabetic controls. ^#^* p* < 0.05, ^##^* p* < 0.01 significant from normal controls.

**Table 2:** Effects of CMPE on lipid profiles of normal and STZ induced diabetic rats.

	Dose (mg/kg)	Triglyceride (mg/dL)	Cholesterol (mg/dL)	HDL-cholesterol (mg/dL)	LDL-cholesterol (mg/dL)
Diabetes control	–	77.97 ± 7.97 ^##^	65.79 ± 8.90 ^##^	23.52 ± 4.26 ^#^	38.64 ± 4.64 ^##^
Diabetes + Tolbutamide	100	75.00 ± 9.74	57.41 ± 3.48 *	24.76 ± 2.32	36.46 ± 2.71
Diabetes + CMPE	150	54.87 ± 10.62 *	52.63 ± 9.68 *	27.09 ± 5.81 *	34.0 6± 4.64 *
Diabetes + CMPE	300	45.14 ± 9.74 **	51.47 ± 8.51 *	24.46 ± 6.97	24.11 ± 4.64 **
Normal control	–	58.41 ± 6.20	50.70 ± 4.64	28.64 ± 4.26	28.25 ± 5.81
Normal + CMPE	150	63.72 ± 9.74	47.60 ± 4.26	26.32 ± 2.32	28.25 ± 1.94
Normal + CMPE	300	60.18 ± 6.20	46.83 ± 4.64	29.03 ± 2.71	28.64 ± 4.26

*n* = 6, data were expressed as mean ± SEM (standard error of the mean). * *p* < 0.05, ** *p* < 0.01 significant from diabetic controls. ^#^* p* < 0.05, ^##^* p* < 0.01 significant from normal controls.

### 2.5. Effects on Malondialdehyde (MDA) Levels in Tissues of Normal and Diabetic Rats

In diabetes, chronic hyperglycemia induces carbonyl stress, which in turn can lead to increased lipid peroxidation [22]. The increased lipid peroxidation can induce oxidative damage by increasing levels of peroxy radicals and hydroxyl radicals [23]. In this study, STZ induction significantly increased hepatic, renal, cordis and splenic levels of MDA, the most commonly used indicator of lipid peroxidation (Table 3). In normal rats, dosage of 150 and 300 mg/kg CMPE caused no significant changes of MDA levels in all tissues tested, compared to normal controls. In diabetic rats, dosages of 300 and 150 mg/kg CMPE exhibited very significantly decreased MDA levels in spleen tissues compared to diabetic control groups. While in kidney and heart tissues, no significant changes of MDA levels were observed in diabetic rats. It was reported that the phenolic extracts from chestnut spiny burs could prevent STZ induced pancreatic β-cell (Rin-5F) death and increase cell viability by protecting DNA from oxidative damage and by enhancing the natural antioxidant system to lower MDA levels in cells [10]. The present *in vivo* study indicated that even CMPE could protect pancreatic β-cells form damage of STZ induction *in vivo*, it possessed marginal effects of protection on pancreas *in vitro*. However, the effects of CMPE and tolbutamide on the spleen MDA levels are very interesting and worthy of further studiy, since the spleen is considered to be involved in the autoimmune pathogenesis of diabetes [24,25].

**Table 3:** Effects of CMPE on MDA levels in tissues of normal and STZ induced diabetic rats.

	Dose (mg/kg)	Liver (mg/g prot)	Kidney (mg/g prot)	Heart (mg/g prot)	Spleen (mg/g prot)
Diabetes control	–	24.68 ± 2.11 ^##^	40.61 ± 5.81 ^##^	37.55 ± 5.58 ^##^	32.65 ± 3.14 ^##^
Diabetes + tolbutamide	100	20.80 ± 2.93 *	46.78 ± 3.34	36.62 ± 4.13	20.53 ± 4.02 **
Diabetes + CMPE	150	22.62 ± 2.53	46.46 ± 5.31	32.38 ± 6.41	24.10 ± 5.15 **
Diabetes + CMPE	300	22.01 ± 0.08 *	43.74 ± 6.95	33.83 ± 3.67	23.60 ± 4.31 **
Normal control	–	17.46 ± 1.6	15.11 ± 1.90	20.29 ± 1.72	26.87 ± 6.15
Normal + CMPE	150	17.94 ± 2.02	16.63 ± 2.42	20.42 ± 1.59	24.62 ± 4.43
Normal + CMPE	300	17.30 ± 1.69	16.11 ± 2.18	22.03 ± 2.67	25.27 ± 6.36

*n* = 6, data were expressed as mean ± SEM (standard error of the mean). * *p* < 0.05, ** *p* < 0.01 significant from diabetic controls. ^##^* p* < 0.01 significant from normal controls.

### 2.6. Effects on Glutathione (GSH) Levels in Tissues of Normal and Diabetic Rats

GSH was usually regarded as another indicator of the health of the antioxidant defence system, and a sharp reduction of GSH levels can usually be observed in diabetic rats [26]. However, in the present study, a marked elevated level of GSH in kidney, liver, heart and spleen was observed in diabetic rats compared to normal rats. As illustrated in Table 4, a significant reduction in the level of GSH was observed almost in all tissues tested of CMPE treated diabetic rats, especially at dose of 300 mg/kg. Tolbutamide also caused a significant reduction in the level of GSH in kidney, heart and spleen of diabetic rats. Similar results were found in *Cydonia oblonga* Mill. leaves and *Allium porrum* L. bulbs extract, which also exhibited hypoglycemic effects but caused a decrease of GSH levels in tissues [6]. The decrease of GSH levels could be the result of decreased synthesis, or increased degradation of GSH by oxidative stress in diabetes [27].

**Table 4:** Effects of CMPE on GSH levels in tissues of normal and STZ induced diabetic rats.

	Dose (mg/kg)	Liver (mg/g prot)	Kidney (mg/g prot)	Heart (mg/g prot)	Spleen (mg/g prot)
Diabetes control	–	114.6 ± 4.0 ^##^	93.6 ± 5.8 ^#^	114.8 ± 21.1 ^##^	102.6 ± 4.2 ^#^
Diabetes + tolbutamide	100	119.8 ± 13.6	69.7 ± 6.6 **	137.8 ± 23.6	90.8 ± 4.2 **
Diabetes + CMPE	150	116.2 ± 16.8 *	77.1 ± 5.8 **	111.6 ± 17.0 *	67.0 ± 6.0 **
Diabetes + CMPE	300	102.1 ± 6.3 **	80.2 ± 6.8 **	96.1 ± 8.0 **	64.7 ± 8.4 **
Normal control	–	68.4 ± 4.4	81.0 ± 4.1	67.6 ± 4.1	84.3 ± 15.4
Normal + CMPE	150	64.4 ± 5.0	86.2 ± 11.1	74.8 ± 12.3	79.8 ± 7.1
Normal + CMPE	300	59.0 ± 1.2 ^##^	85.9 ± 5.4	63.8 ± 6.3	86.2 ± 6.7

*n* = 6, data were expressed as mean ± SEM (standard error of the mean). * *p* < 0.05, ** *p* < 0.01 significant from diabetic controls. ^#^* p* < 0.05, ^##^* p* < 0.01 significant from normal controls.

## 3. Experimental

### 3.1. Plant Samples, Animals and Reagents

The Chinese chestnut burs were harvested at a chestnut plantation in Qianxi, Hebei Province of China at the beginning of the harvest season of 2008, and authenticated by Dr Yujun Liu, Beijing Forestry University. The burs were air-dried for about 4 days at about 25 °C till equilibrium humidity was reached, ground and kept under darkness for further use. All chemicals were purchased from Sigma-Aldrich, Inc. unless otherwise specified. Male Wistar-albino rats were purchased from the Laboratory Animal Center of the Academy of Military Medical Sciences, China, and were housed in a room under controlled conditions with temperature maintained at 23 ± 2 °C, on a 12 h light: 12 h dark cycle. The animals were fed on pelleted food, and tap water was available *ad libitum*. Throughout the experiments, animals were monitored and maintained in accordance with the ethical recommendations and guidelines for the care of laboratory animals. Prior to the experiments, rats were fed with standard rodent food for 1 week in order to adapt to the laboratory conditions. All animals were fasted overnight (12 h) before experiments, but free access to water was available.

### 3.2. Preparation of Phenolic Extracts of C. mollissima (CMPE) Spiny Burs

Air-dried chestnut burs (1 kg) were extracted three times using 50% aqueous EtOH (2 L) in a shaking constant-temperature water bath at 80 °C for 1 h each time. The resulting slurries were centrifuged at 5,000 g for 10 min (model GL 10MD, Changsha, China), and the supernatant was collected, combined and evaporated till 20% volume was left, and subsequently replenished with distilled water to the initial volume. After replenishment, the supernatant was centrifuged at 5,000 g for 10 min, and the supernatant was subjected to column chromatography (600 mm × 60 mm i.d.) on a HPD 100 macroporous resin and eluted with 2,000 mL of 50% (v/v) ethanol/water after washing with 2,000 mL distilled water. The 50% ethanol elution was evaporated and lyophilized to derive the phenolic extracts of *C. mollissima* (CMPE). The total tannins and polyphenols content in CMPE were 622.9 mg and 836.4 mg GAE, respectively (Gallic Acid Equivalent)/g, determined according to the method described in literature [9]). Finally, the CMPE was stored at −20 °C for further analysis. Prior to oral administration to the experimental animals, CMPE was suspended in distilled water.

### 3.3. Determination of the Blood Glucose Levels

Fasting blood samples were collected from the tip of tail veins of the animals at the defined times in the protocol. Whole blood was used to determine the glucose concentration in a glycometer immediately after collection, using GLUCOCARD Test Strip II (ARKRAY Factory, Inc. Japan) based on the glucose oxidase method.

### 3.4. Induction of Diabetic Rats

Male Wistar-albino rats (180–220 g) were rendered diabetic by two intraperitoneal (i.p.) injections of streptozotocin (STZ) freshly dissolved in 0.1 M citrate buffer (pH 4.5) at a dose of 60 mg/kg to 16 h fasted rats with an interval of 5 days [28]. Aged matched normal animals receiving an injection of an equivalent volume of 0.1 M citrate buffer (pH 4.5) comprised a non-diabetic control group. Diabetes was confirmed by the presence of hyperglycemia, polyphagia, polydipsia, polyuria and body weight loss. Seven days afterwards, fasting blood glucose levels were measured and animals with blood glucose concentration above 250 mg/dL were considered to be diabetic and selected for the subsequent experiments.

### 3.5. Effects of CMPE on Diabetic and Normal Rats

STZ-induced diabetic rats were randomly allocated and similarly grouped into four groups of six animals: diabetic group (treated with normal saline, 5 mL/kg body wt., *p.o.*), 100 mg/kg body wt. (*p.o*.) tolbutamide treated diabetic group, 150 mg/kg body wt. (*p.o.*) CMPE treated diabetic group, 300 mg/kg body wt. (*p.o.*) CMPE treated diabetic group. Body weight matched normal rats were also randomly allocated into three groups of six animals each: normal control group (treated with normal saline, 5 mL/kg body wt., *p.o.*), 150 mg/kg body wt. (*p.o.*) CMPE treated normal group, and 300 mg/kg body wt. (*p.o.*) CMPE treated normal group. Test samples of CMPE were orally administrated twice a day for 12 consecutive days. Fasting blood glucose levels were determined on the 3rd, 6th, 9th and 12th day after and before (1st day) the administration of the test samples. The effect of each sample on body weight was also monitored at the same time. On 12th day, all animals were sacrificed and then the kidney, spleen, liver, and heart of each animal were removed for measurement of tissue MDA and GSH levels. Also, blood was collected from abdominal aorta, and serum triglyceride, total cholesterol, and HDL cholesterol and LDL cholesterol levels were spectrophotometrically measured using appropriate kits (Biosino Bio-technology and Science Inc., China).

### 3.6. Measurement of MDA Concentration in Liver, Kidney, Spleen and Heart Tissues

After removal the liver, spleen, heart and kidney tissue samples were immediately washed with normal saline, blotted dry and weighted exactly. Then, tissues were made into 5% homogenate in ice-cold saline. A supernatant was obtained from tissue homogenate by centrifugalization (1,000 × *g*, 4 °C, 10 min). The MDA concentration was measured based on the thibabituric acid (TBA) reaction method according to the kit handbook guidelines (Nanjing Jiancheng Bioengineering Institute, China). Briefly, 20% trichloroacetic acid (1.0 mL) and 1% TBA reagent (1.0 mL) were added to supernatant (100 μL), then mixed and incubated at 95 °C for 80 min. After cooling on ice, samples were centrifuged at 1,000 × *g* for 20 min and the absorbance of the supernatant was measured at 532 nm. The results were expressed as MDA equivalents using tetraethoxypropane as standard. The protein concentration in tissues was measured using BCA protein assay kit (Mbchem™, China).

### 3.7. Non-Protein Sulphydryl Groups in Liver, Kidney, Spleen and Heart Tissues

After excision, livers, kidneys, spleen and heart tissues were homogenized in 8.0 mL of 0.02 M EDTA in an ice bath. Aliquots of 5.0 mL of the homogenates were mixed in 15.0 mL of 50% trichloroacetic acid. Afterwards, the tubes were centrifuged at 3,000 × *g* for 15 min, and 2.0 mL of supernatant was mixed with 4.0 mL of 0.4 M Tris buffer. Then, 0.1 mL Ellman’s reagent was added and the absorbance at 412 nm against a reagent blank with no homogenate was recorded after shaken. Results were expressed as μmol GSH/g prot.

### 3.8. Statistical Analysis

The data were expressed as means ± SEM (standard error of the mean). Statistical analysis between the treatments and the control were performed using Student’s paired *t*-test, repeated measure and one-way ANOVA followed by Tukey post hoc test. A difference in the mean values of *p* < 0.05 was considered to be statistically significant.

## 4. Conclusions

The present study evaluated the hypoglycemic and hypolipidemic effects of polyphenol extracts of *Castanea mollissima* Blume spiny burs (CMPE) in normal and STZ induced diabetic rats. It illustrated that after oral administration of CMPE at a dose of 150 mg/kg for 12 consecutive days, the fasting glucose levels in STZ-induced diabetic rats were significantly decreased and at a dose of 300 mg/kg, the serum glucose, triglyceride, total cholesterol, LDL-cholesterol levels were also significantly reduced. CMPE administration could also significantly decrease the MDA and GSH levels in spleen and liver tissues. In normal rats, no significant differences were found in serum glucose levels, serum lipid profiles and MDA and GSH levels in tissues. As a conclusion, the present study confirmed for the first time the *in vivo* hypoglycemic and hypolipidemic effects of CMPE. The observed hypoglycemic and hypolipidemic effects of CMPE on diabetic rats extend our knowledge about the potential bioactivities and applications in the pharmaceutical and food processing industries of polyphenols abundant in the chestnut burs. It could be used by diabetic patients to decrease the complications of diabetes. However, it was also found that dosage of 300 mg/kg CMPE caused a significant body weight gain loss in both normal and diabetic rats. Therefore, further studies are necessary to determine the exact nature of the active principles, the mechanism of action and to assess the safety of CMPE.
